# Functional evaluation of *TERT-CLPTM1L* genetic variants associated with susceptibility of papillary thyroid carcinoma

**DOI:** 10.1038/srep26037

**Published:** 2016-05-17

**Authors:** Minghua Ge, Meng Shi, Changming An, Wenjun Yang, Xilin Nie, Jian Zhang, Zheng Lv, Jinliang Li, Liqing Zhou, Zhongli Du, Ming Yang

**Affiliations:** 1 Department of Head and Neck Surgery, Zhejiang Province Cancer Hospital, Hangzhou, Zhejiang Province, China; 2 College of Life Science and Technology, Beijing University of Chemical Technology, Beijing, China; 3 Department of Head and Neck Surgical Oncology, Cancer Hospital, Chinese Academy of Medical Sciences, Beijing, China; 4 Key Laboratory of Fertility Preservation and Maintenance (Ministry of Education), Ningxia Medical University, Yinchuan, Ningxia, China; 5 Department of Clinical Laboratory, Qilu Hospital of Shandong University, Jinan, Shandong Province, China; 6 Cancer Center, The First Affiliated Hospital of Jilin University, Changchun, Jilin Province, China; 7 Department of Radiation Oncology, Huaian No. 2 Hospital, Huaian, Jiangsu Province, China; 8 Department of Hematology, National Center for Clinical Laboratories and Beijing Hospital, Beijing, China; 9 Shandong Key Laboratory of Radiation Oncology, Cancer Research Center, Shandong Cancer Hospital and Institute, Jinan, Shandong Province, China

## Abstract

TERT is the catalytic subunit of telomerase which plays an essential part in cellular immortality by maintaining telomere integrity. TERT is commonly over-expressed in human malignancies, indicating its key role in cell transformation. The chromosome 5p15.33 *TERT-CLPTM1L* region has been associated with susceptibility of multiple cancers via a genome-wide association approach. However, the involvement of this locus in papillary thyroid carcinoma (PTC) etiology is still largely unknown. We analyzed 15 haplotype-tagging single nucleotide polymorphisms (htSNPs) of the *TERT*-*CLPTM1L* region in a two stage case-control design. After genotyping 2300 PTC patients and frequency-matched 2300 unaffected controls, we found that *TERT* rs2736100 genetic variant is significantly associated with elevated PTC risk. *Ex vivo* reporter gene assays indicated that the PTC susceptibility rs2736100 polymorphism locating in a potential *TERT* intronic enhancer has a genotype-specific effect on *TERT* expression. Correlations between rs2736100 genotypes and tissue-specific *TERT* expression supported the regulatory function of this genetic variant *in vivo*. Our data demonstrated that the functional *TERT* rs2736100 SNP as a novel genetic component of PTC etiology. This study, together with recent studies in other cancers, unequivocally establishes an essential role of TERT in cancers.

Thyroid carcinoma is the most common endocrine malignancy and showed quickly increased incidence over last two decades. According to the Chinese Cancer Registry, the incidence of thyroid carcinoma is 6.6 per 100,000 individuals in China^1^,^2^. Papillary thyroid carcinoma (PTC), named for their papillary histological architecture, accounts for about eighty percent of all thyroid carcinomas. Ionizing radiation, nodular disease of the thyroid and family history account for known risk factors of PTC currently^3^. However, only a portion of exposed individuals develop PTC, suggesting that genetic factors may also impact thyroid malignant transformation^4^.

Accumulated evidences demonstrated that the chromosome 5p15.33 region (*TERT-CLPTM1L*) is a common susceptibility locus of multiple cancers. Genome-wide association studies (GWAS) declared that independent susceptibility single nucleotide polymorphisms (SNPs) in 5p15.33 were identified in different malignancies, including lung cancer^5^,^6^,^7^,^8^,^9^,^10^, melanoma^11^, nonmelanoma skin cancer^11^,^12^, glioma^13^, bladder cancer^14^, pancreatic cancer^15^, testicular germ cell cancer^16^, estrogen-negative breast cancer^17^, ovarian cancer^18^ and prostate cancer^19^. Therefore, it is plausible that several functional DNA elements might exist in the region and influence cancer etiology. There are two known oncogenes, *TERT* and *CLPTM1L*, in the locus. Activated *TERT* (telomerase reverse transcriptase) transcription enhances telomerase activities and accelerates malignant transformation^20^,^21^. In lung cancer, oncogene *CLPTM1L* (cleft lip and palate-associated transmembrane 1 like protein) plays an a protumorigenic role and is critical for Ras-driven lung cancers^22^,^23^,^24^. In pancreatic cancer, *CLPTM1L* functions as a growth-promoting gene and its overexpression may lead to an abrogation of normal cytokinesis and enhance aneuploidy in pancreatic cancer cells^22^,^23^,^24^.

Considering the impacts of the 5p15.33 *TERT-CLPTM1L* locus on PTC susceptibility is still largely unknown, we examined the associations between 15 haplotype-tagging SNPs (htSNP) covering the entire *TERT-CLPTM1L* locus and PTC risk in three large independent case-control studies. To investigate the biological function of the PTC susceptibility *TERT* rs2736100 SNP, we examined impacts of its genotypes on *TERT* expression *ex vivo* and *in vivo*.

## Material and Methods

### Study subjects

A total of three case-control sets were included in the current study. (i) Zhejiang set: 500 PTC cases from Zhejiang Province Cancer Hospital (Hangzhou, Zhejiang Province, China) and sex- and age-matched 500 controls. (ii) Jiangsu set: 1000 cases with PTC from Huaian No. 2 Hospital (Huaian, Jiangsu Province, China) and sex- and age-matched (±5 years) 1000 healthy controls. (iii) Jilin set: 800 PTC patients from The First Affiliated Hospital of Jilin University (Changchun, Jilin Province, China) and 800 sex- and age-matched healthy controls. Sixty pairs of PTC specimens and thyroid normal tissues adjacent to the tumors were obtained from surgically removed specimens of patients in Zhejiang Province Cancer Hospital and Huaian No. 2 Hospital. All individuals were ethnic Han Chinese. The detailed information on subject recruitments can be found in Table 1. This study was approved by the institutional Review Boards of Zhejiang Province Cancer Hospital, Huaian No. 2 Hospital and The First Affiliated Hospital of Jilin University. At recruitment, the written informed consent was obtained from each subject. The methods were carried out in accordance with the approved guidelines.

### SNP selection and genotyping

An htSNP approach was used to investigate genetic polymorphisms in the *TERT-CLPTM1L* locus globally (a 91716 bp region of chromosome 5p15.33)^25^,^26^,^27^. HapMap SNPs which have been genotyped among Han Chinese and Japanese populations (HapMap Rel 21, NCBI B36) with a minor allele frequency >5% were included in htSNP selection. A total of 15 htSNPs were chosen in a 95716 bp region (the 91716 bp *TERT-CLPTM1L* locus and 2 kb up-stream plus 2 kb down-stream regions of the locus). The selection criteria included the sample size inflation factor, Rh^2^, of ≥0.8 and a block-by-block method using Haploview 4.2 software (Supplementary Table 1). All htSNPs were genotyped through the MassArray system (Sequenom Inc., San Diego, California, USA). A 5% blind, random DNA samples was analyzed in duplicates and the reproducibility was 99%. To reduce the costs of the study, we genotyped the *TERT* rs2736100 T > G SNP in two validation sets using the PCR-based restriction fragment length polymorphism (RFLP) as described in Supplementary Table 2. A 5% samples were genotyped by two investigators and the reproducibility was 98.5%.

### Dual-luciferase reporter gene assays

The intron 2 region of *TERT* (including the rs2736100 flanking region) was amplified with human genomic DNA from healthy control individuals carrying either *TERT* rs2736100 TT genotype or rs2736100 GG genotype. Specific PCR primer pairs with the *Kpn*I and *Xho*I restriction sites were showed in Supplementary Table 3. The PCR products were digested and ligated into an appropriately digested pGL3-Basic vector. The resultant *TERT* reporter gene plasmids were designated pTERT-T or pTERT-G, which were only different at the rs2736100 polymorphic site. Sanger sequencing of the insertions confirmed the orientation and integrity of the two constructs.

Both reporter gene constructs (pGL3-Basic, pTERT-T, or pTERT-G) and pRL-SV40 (Luciferase Assay System; Promega) were transfected into PTC cell line BCPAP cells or HEK293 cells. As previously described, dual luciferase activities were determined at 48 h after transfection^28^. For each plasmid construct, three independent transfection experiments were performed, and each was done in triplicates.

### Real-time qPCR of TERT mRNA

Total cellular RNA was isolated from sixty pairs of PTC specimens and normal tissues adjacent to the tumors with TRIzol Reagent (Invitrogen) and converted to cDNA using the PrimeScript RT Master Mix (TaKaRa). *TERT* mRNA expression in tissues was analyzed using the TaqMan real-time qPCR method. Relative gene expression quantization for *TERT* (ABI, Assay ID Hs00972656_m1) was calculated using *β-actin* (ABI, Assay ID 4333762T) as an internal reference gene was carried out using the ABI 7500 real-time PCR system in triplicates.

### Statistics

The Pearson chi-square test was used to examine selected characteristics between PTC cases and controls for categorical variables. The associations between *TERT-CLPTM1L* genotypes and PTC risk were estimated by odds ratios (ORs) and their 95% confidence intervals (CIs) computed by logistic regression models. All ORs were adjusted for age or sex, where it was appropriate. One-way ANOVA was used for the correlations between genotypes of rs2736100 and *TERT* mRNA expression. A *P* value of less than 0.05 was used as the criterion of statistical significance. All statistical tests were two-sided and performed using SPSS 16.0 (SPSS Inc.).

## Results

Table 2 showed genotype distributions of 15 SNPs in the *TERT*-*CLPTM1L* loci in the Zhejiang discovery set. All observed genotype frequencies in both PTC patients and controls conform to Hardy-Weinberg equilibrium (all *P* > 0.05). Among the 15 SNPs, frequencies of rs2736100 genotypes among cases differed significantly from those among healthy controls (*P* < 0.05). In details, rs2736100 genetic variant was associated with significantly elevated PTC risk (allelic OR = 1.39, 95% CI = 1.16–1.66, *P* = 7.0 × 10^−6^) (Table 2). There were no statistically significant associations between other 14 SNPs and PTC risk (all *P* > 0.05) (Table 2), we did not examine these SNPs in the next analyses.

Logistic regression analyses showed that the rs2736100 G allele was a risk allele. Subjects having the TG genotype had an OR of 1.34 (95% CI = 1.01–1.79, *P* = 0.047) for developing PTC compared with subjects having the TT genotype. It was observed that the odds of having the rs2736100 GG genotype in cases was 1.36 (95% CI = 1.14–1.62, *P* = 7.4 × 10^−4^) compared with the TT genotype. In Jiangsu validation set, a significantly increased OR was also associated with the rs2736100 GT or GG genotype (OR = 1.44, 95% CI = 1.18–1.76, *P* = 0.003) or (OR = 1.43, 95% CI = 1.26–1.62, *P* = 3.8 × 10^−6^). Moreover, the significant association between rs2736100 SNP and PTC risk were also observed in Jilin validation set (Table 3). Individuals with rs2736100 GG genotype showed significantly increased PTC risk compared with those with rs2736100 TT genotype (OR = 1.18, 95% CI = 1.02–1.37, *P* = 0.025). However, rs2736100 GT genotype was not significantly associated with PTC risk (OR = 1.05, 95% CI = 0.82–1.34, *P* = 0.695) in Jilin set. The PTC risk associated with the rs2736100 genetic variant was further examined by stratifying for age and sex using the combined data of three case-control sets (Table 4). Significant associations between rs2736100 TG or GG genotype and PTC risk were observed in all stratified groups (all *P* < 0.05).

**Figure 1:**
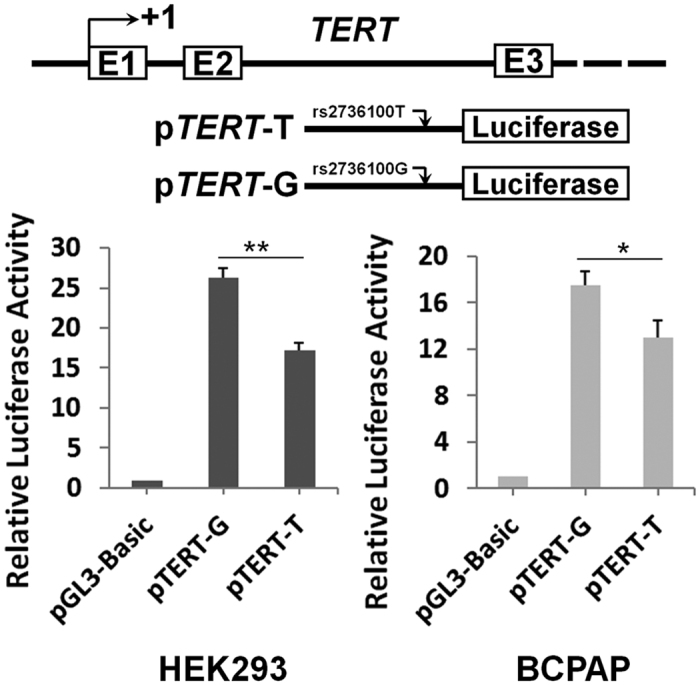
Transient luciferase reporter gene expression assays with constructs containing different rs2736100 allele of the *TERT* intron 2 region in HEK293 cells (**A**) or BCPAP cells (**B**). pRL-SV40 were cotransfected with these contructs to standardize transfection efficiency. Fold-changes were detected by defining the luciferase activity of cells co-transfected with pGL3-basic as 1. All experiments were performed in triplicates in three independent transfection experiments and each value represents mean ± SD. Compared with pGL3-Basic transfected cells, *P < 0.05; **P < 0.01.

Since the rs2736100 variant locates in the *TERT* intron 2 region, we investigated the impacts of this polymorphism on *TERT* gene expression via dual-luciferase reporter gene assays (Fig. 1). We found that the intron 2 segment containing the rs2736100 flanking sequence showed enhancer activities in HEK293 cells or BCPAP PTC cells. Moreover, the *TERT* rs2736100G allelic reporter construct (pTERT-G) showed significantly higher luciferase activities compared to the rs2736100T allelic reporter construct (pTERT-T) in HEK293 cells (*P* < 0.01) or BCPAP PTC cells (*P* < 0.05) (Fig. 1).

**Figure 2:**
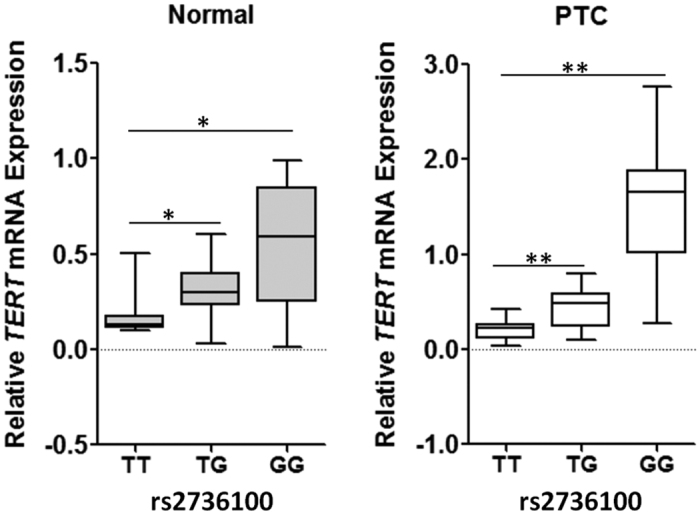
*TERT* mRNA expression in normal and cancerous thyroid tissues grouped by rs2736100 genotypes. The expression of individual *TERT* mRNA was calculated relative to expression of *β-actin* using the 2^−dCt^ method. **P < 0.01.

We next examined the allele-specific effect of rs2736100 polymorphism on *TERT* gene expression in thyroid tissue specimens. Interestingly, significant up-regulation of *TERT* in PTC tissues was observed compared with normal tissues (*P* = 0.0003). We found that subjects with the rs2736100 GG or GT genotype had significantly lower *TERT* mRNA levels (mean ± SE) than those with the TT genotype in normal thyroid tissues (0.538 ± 0.078 [the rs2736100 GG genotype, *n* = 16] or 0.322 ± 0.023 [the rs2736100 GT genotype, *n* = 27] vs. 0.164 ± 0.024 [the rs2736100 TT genotype, *n* = 17], both *P* < 0.05). As shown in Fig. 2B, similar results were found in PTC tissues (1.550 ± 0.188 [the rs2736100 GG genotype, *n* = 16] or 0.441 ± 0.036 [the rs2736100 GT genotype, *n* = 27] vs. 0.214 ± 0.026 [the rs2736100 TT genotype, *n* = 17], both *P* < 0.01).

## Discussion

In this study, we systematically evaluated PTC susceptibility genetic variants in the *TERT*-*CLPTM1L* locus and their regulatory role in *TERT* gene expression *ex vivo* and *in vivo*. In the discovery case-control set, we identified one PTC susceptibility genetic variant (rs2736100) after genotyping 15 *TERT*-*CLPTM1L* htSNPs. The significant association between *TERT* rs2736100 and PTC was validated in two validation case-control sets. *Ex vivo* luciferase gene assays demonstrated that the PTC susceptibility rs2736100 polymorphism locates in a potential *TERT* intronic enhancer and has a genotype-specific impact on *TERT* expression. Additionally, correlations between rs2736100 genotypes and tissue-specific *TERT* gene expression levels supported the regulatory function of this genetic variant *in vivo*.

TERT is the catalytic subunit of telomerase which that plays a essential part in cellular immortality by maintaining telomere length at the end of chromosomes^29^,^30^. TERT is well-known to be over-expressed in many human malignancies, indicating its key role in transformation of human normal cells^31^. In line with this, transgenic mice with induced *TERT* expression showed significantly increased development of tumors^32^,^33^. High TERT expression and telomerase activity have been found in thyroid cancers, particularly in the advanced forms of the disease^34^,^35^. Additionally, highly prevalent *TERT* promoter mutations have been repeatedly found in PTC, which highlighting the importance of in etiology of PTC^36^,^37^,^38^.

Although the *TERT* rs2736100 SNP were repeatedly identified as a susceptibility polymorphisms in many cancers^4^,^5^,^6^,^7^,^8^,^9^,^10^,^11^,^12^,^13^,^14^,^15^,^16^, its role in PTC etiology is still largely unknown even after several GWAS of thyroid cancer publised^39^,^40^,^41^. To the best of our knowledge, this is the first study to examine the association between the *TERT* rs2736100 polymorphism and PTC risk. We believe that the association between the rs2736100 SNP and increased PTC risk is biologically plausible since the PTC susceptibility rs2736100 G allele showed consistently higher oncogene *TERT* gene expression than T allele.

In all, we identified the functional *TERT* rs2736100 genetic polymorphism as a novel genetic component of the PTC etiology in Chinese populations. This study, together with recent studies in other cancers, unequivocally establishes an important role of *TERT* SNPs in cancer development, especially human thyroid cancer. However, further investigations in additional ethnic populations are desirable to validate our observations.

## Additional Information

**How to cite this article**: Ge, M. *et al*. Functional evaluation of *TERT-CLPTM1L* genetic variants associated with susceptibility of papillary thyroid carcinoma. *Sci. Rep.* **6**, 26037; doi: 10.1038/srep26037 (2016).

## Supplementary Material

Supplementary Information

## Figures and Tables

**Table 1:** Distribution of selected characteristics among PTC cases and controls.

**Variable**	**Zhejiang case-control set (Discovery set)**			**Jiangsu case-control set (Validation set 1)**			**Jilin case-control set (Validation set 2)**		
	**Cases**	**Controls**	***P*****-value**^**a**^	**Cases**	**Controls**	***P*****-value**^**a**^	**Cases**	**Controls**	***P*****-value**^**a**^
	**No. (%)**	**No. (%)**		**No. (%)**	**No. (%)**		**No. (%)**	**No. (%)**	
	500	500		1000	1000		800	800	
Sex			0.560			0.223			0.774
Male	122(24.4)	130(26.0)		251(25.1)	275(27.5)		200(25.0)	205(25.6)	
Female	378(75.6)	370(74.0)		749(74.96)	725(72.5)		600(75.0)	595(74.4)	
Age (year)^2^			0.486			0.823			0.453
≤46(or 48)	258(51.6)	269(53.8)		526(52.6)	521(52.1)		411(51.4)	426(53.2)	
>46(or 48)	242(48.4)	231(46.2)		474(47.4)	479(47.9)		389(48.6)	374(46.8)	

Note: PTC, papillary thyroid carcinoma.

^1^Two-sided χ^2^ test.

^2^Median ages of cases for Zhejiang case-control set, Jiangsu case-control set and Jilin case-control set are 46, 48 and 48 years.

**Table 2:** Associations between candidate SNPs in the *TERT-CLPTM1L* locus and risk of PTC in Zhejiang case-control set.

**No.**	**rs ID**	**Position**	**Base change**	**MAF**^1^	**Genotype (500 cases and 500 controls)**				***P*****-value**^3^
					**Common**^2^	**Heterozygous**^2^	**Rare**^2^	**OR(95% CI)**^3^	
1	rs2853691	1305950	T > C	0.184	66.9/67.2	28.9/28.8	2.1/2.0	1.02(0.81–1.28)	0.871
2	rs2736122	1310621	G > A	0.045	91.9/91.0	8.1/9.0	0/0	0.91(0.59–1.40)	0.656
3	rs2075786	1319310	A > G	0.196	65.3/64.3	31.4/32.1	3.3/3.6	0.96(0.77–1.20)	0.700
4	rs4246742	1320356	T > A	0.425	32.6/31.9	50.1/51.2	17.3/16.9	1.01(0.84–1.20)	0.949
5	rs4975605	1328528	C > A	0.145	73.1/72.2	25.5/26.6	1.4/1.2	1.03(0.80–1.32)	0.841
**6**	**rs2736100**	**1339516**	**T > G**	**0.413**	**26.6/35.2**	**48.0/47.0**	**25.4/17.8**	**1.39(1.16–1.66)**	**2.7 × 10**^**−4**^
7	rs2853676	1341547	C > T	0.134	78.1/75.1	19.5/23.1	2.4/1.8	0.90(0.69–1.17)	0.645
8	rs2736098	1347086	C > T	0.335	42.4/43.3	46.9/46.5	10.7/10.2	0.97(0.81–1.17)	0.741
9	rs2853668	1353025	G > T	0.260	54.0/54.6	39.1/38.8	6.9/6.6	0.99(0.81–1.20)	0.893
10	rs2735845	1353584	C > G	0.322	47.7/47.7	41.2/40.2	11.1/12.1	0.98(0.81–1.18)	0.811
11	rs6554759	1370102	A > G	0.154	72.2/71.9	25.1/25.5	2.7/2.6	0.99(0.78–1.27)	0.951
12	rs451360	1372680	C > A	0.177	71.7/70.1	25.0/26.1	3.3/3.8	0.92(0.73–1.17)	0.512
13	rs380286	1373247	G > A	0.156	70.0/71.4	26.9/26.1	3.1/2.5	1.08(0.85–0.37)	0.543
14	rs402710	1373722	C > T	0.314	45.6/47.6	42.9/42.1	11.5/10.3	1.08(0.89–0.30)	0.444
15	rs452932	1383253	T > C	0.196	63.8/65.1	31.3/30.7	4.9/4.2	1.06(0.86–1.32)	0.577

Note: PTC, papillary thyroid carcinoma; MAF, minor allele frequency; OR, odds ratios; 95%CI, 95% confident intervals.

^1^MAF in healthy controls.

^2^% of case/% of control.

^3^Allelic OR calculated by logistic regression.

**Table 3:** Genotype frequencies of rs2736100 T > G SNP in the *TERT-CLPTM1L* locus among cases and controls and their association with PTC risk.

**Studies**	**rs2736100 T > G**				
	**Genotypes**	**Cases No. (%)**	**Controls No. (%)**	**OR**^**2**^ **(95% CI)**	***P*****-value**
		*n* = 500	*n* = 500		
Zhejiang set	TT	133(26.6)	176(35.2)	Reference	
	TG	240(48.0)	235(47.0)	1.34(1.01–1.79)	0.047
	GG	127(25.4)	89(17.8)	1.36(1.14–1.62)	7.4 × 10^−4^
	*n* = 1000	*n* = 1000			
Jiangsu set	TT	293(29.3)	398(39.8)	Reference	
	TG	476(47.6)	448(44.8)	1.44(1.18–1.76)	0.003
	GG	231(23.1)	154(15.4)	1.43(1.26–1.62)	3.8 × 10^−6^
		*n* = 800	*n* = 800		
Jilin set	TT	218(27.3)	301(37.6)	Reference	
	TG	377(47.1)	373(46.6)	1.05(0.82–1.34)	0.695
	GG	205(25.6)	126(15.8)	1.18(1.02–1.37)	0.025
		*n* = 2300	*n* = 2300		
Pooled	TT	644(28.0)	875(38.0)	Reference	
	TG	1093(47.5)	1056(45.9)	1.41(1.23–1.60)	4.9 × 10^−6^
	GG	563(24.5)	369(16.0)	1.44(1.33–1.56)	8.5 × 10^−8^

Note: PTC, papillary thyroid carcinoma; OR, odds ratio; CI, confidence interval.

^1^Data were calculated by logistic regression with adjustment for age and sex.

**Table 4:** Risk of PTC associated with rs2736100 T > G genotypes by age and sex.

**Variable**	**rs2736100 T > G**						
	**TT**^1^	**TG**^1^	**OR**^2^ **(95% CI)**	***P*****-value**	**GG**^1^	**OR**^2^ **(95% CI)**	***P*****-value**
Sex							
Male	156/245	275/277	1.57(1.21–2.03)	0.001	142/88	1.59(1.35–1.88)	4.3 × 10^−6^
Female	488/630	818/779	1.35(1.16–1.58)	0.001	421/281	1.39(1.27–1.53)	1.3 × 10^−6^
Age (year)							
≤47	314/446	536/532	1.43(1.19–1.73)	0.001	287/176	1.52(1.35–1.72)	3.3 × 10^−6^
>47	330/429	557/524	1.38(1.15–1.67)	0.001	276/193	1.36(1.21–1.53)	2.1 × 10^−5^

Note: PTC, papillary thyroid carcinoma; OR, odds ratio; CI, confidence interval.

^1^Number of case patients with genotype/number of control subjects with genotype.

^2^Data were calculated by logistic regression, adjusted for sex and age, where it was appropriate.
