# Supplementary material for: Functional evaluation of TERT-CLPTM1L genetic variants associated with susceptibility of papillary thyroid carcinoma
Source: Sci Rep. 2016 May 17;6:26037. doi: 10.1038/srep26037 (PMC4869017; doi:10.1038/srep26037)
Supplement: Supplementary Information [file srep26037-s1.pdf]

Functional evaluation of *TERT-CLPTM1L* genetic variants associated with susceptibility of papillary thyroid carcinoma

Minghua Ge[1,†,*]; Meng Shi[2,†]; Changming An[3,†]; Wenjun Yang[4]; Xilin Nie[1]; Jian Zhang[5]; Zheng Lv[6]; Jinliang Li[2]; Liqing Zhou[7]; Zhongli Du[8]; Ming Yang[9,*]

Authors' affiliations: [1]Department of Head and Neck Surgery, Zhejiang Province Cancer Hospital, Hangzhou, Zhejiang Province, China; [2]College of Life Science and Technology, Beijing University of Chemical Technology, Beijing, China; [3]Department of Head and Neck Surgical Oncology, Cancer Hospital, Chinese Academy of Medical Sciences, Beijing, China; [4]Key Laboratory of Fertility Preservation and Maintenance (Ministry of Education), Ningxia Medical University, Yinchuan, Ningxia, China; [5]Department of Clinical Laboratory, Qilu Hospital of Shandong University, Jinan, Shandong Province, China; [6]Cancer Center, The First Affiliated Hospital of Jilin University, Changchun, Jilin Province, China; [7]Department of Radiation Oncology, Huaian No. 2 Hospital, Huaian, Jiangsu Province, China; [8]Department of Hematology, National Center for Clinical Laboratories and Beijing Hospital, Beijing, China; [9]Shandong Key Laboratory of Radiation Oncology, Cancer Research Center, Shandong Cancer Hospital and Institute, Jinan, Shandong Province, China.

[†]**Note:** Minghua Ge, Meng Shi and Changming An contributed equally to this work.

***Correspondence to:** Ming Yang, PhD, Professor, Shandong Key Laboratory of Radiation Oncology, Cancer Research Center, Shandong Cancer Hospital and Institute, Jinan 250117, Shandong Province, China. Tel & Fax: 8610-64447747; E-mail: aaryoung@yeah.net; or, Minghua Ge, MD, Professor, Department of Head and Neck Surgery, Zhejiang Province Cancer Hospital, Hangzhou 310022, Zhejiang Province, China. Tel: 86-13605813782; Fax: 86-571-88122508; E-mail: gemingh@163.com.

Supplementary Table 1. HapMap tag-SNPs in *TERT-CLPTM1L* locus with Han Chinese Beijing and Japanese in Tokyo data

Test	Alleles Captured
rs452932	rs27070,rs4975615,rs421629,rs465498,rs4975616,rs31489,rs452384,rs467095,rs466502,rs31484,rs452932
rs6554759	rs10073340,rs1801075,rs6554759
rs451360	rs4635969,rs451360
rs402710	rs402710,rs401681
rs2736122	rs2736122
rs2075786	rs2075786
rs2736100	rs2736100
rs2853691	rs2853691
rs2736098	rs2736098
rs380286	rs380286
rs2853668	rs2853668
rs2735845	rs2735845
rs4246742	rs4246742
rs4975605	rs4975605
rs2853676	rs2853676

Note: SNP, single nucleotide polymorphism.

Supplementary Table 2. Genotyping *TERT* rs2736100 T>G using PCR-based restriction fragment length polymorphism

SNPs	PCR Primers (5'→ 3')	Restriction endonucleases	Restriction fragment length (bp)
rs2736100 (T>G)	F: GCAGGGCGGGGGCAAAGGTA<br>R: CACCCCCCAGCCTGTGTGCTGT	*Rsa*I	TT: 132<br>TG: 132+111+21<br>GG: 111+21

Abbreviations: TERT, Telomerase reverse transcriptase; PCR, polymerase chain reaction; SNP, single nucleotide polymorphisms; bp, base pair.
Note: The mismatch base in PCR primers was underlined.

Supplementary Table 3. PCR Primers used in construction of luciferase reporter plasmids

Plasmid constructs	PCR clone Primers (5'→ 3')
pTERT	F: CGGGGTACCGTGTAATGGCACAATCTCGG<br>R: CCGCTCGAGCAAGCAGAAGGGAGGAAGC

Abbreviations: PCR, polymerase chain reaction.
